# The Application of Imaging Flow Cytometry for Characterisation and Quantification of Bacterial Phenotypes

**DOI:** 10.3389/fcimb.2021.716592

**Published:** 2021-07-21

**Authors:** Ann L. Power, Daniel G. Barber, Sophie R. M. Groenhof, Sariqa Wagley, Ping Liu, David A. Parker, John Love

**Affiliations:** ^1^ Biosciences, College of Life and Environmental Sciences, University of Exeter, Exeter, United Kingdom; ^2^ Shell International Exploration & Production Inc., Westhollow Technology Center, Houston, TX, United States

**Keywords:** imaging flow cytometry, bacteria, phenotypes, cell morphology, persister cells, viable but non culturable cells

## Abstract

Bacteria modify their morphology in response to various factors including growth stage, nutrient availability, predation, motility and long-term survival strategies. Morphological changes may also be associated with specific physiological phenotypes such as the formation of dormant or persister cells in a “viable but non-culturable” (VBNC) state which frequently display different shapes and size compared to their active counterparts. Such dormancy phenotypes can display various degrees of tolerance to antibiotics and therefore a detailed understanding of these phenotypes is crucial for combatting chronic infections and associated diseases. Cell shape and size are therefore more than simple phenotypic characteristics; they are important physiological properties for understanding bacterial life-strategies and pathologies. However, quantitative studies on the changes to cell morphologies during bacterial growth, persister cell formation and the VBNC state are few and severely constrained by current limitations in the most used investigative techniques of flow cytometry (FC) and light or electron microscopy. In this study, we applied high-throughput Imaging Flow Cytometry (IFC) to characterise and quantify, at single-cell level and over time, the phenotypic heterogeneity and morphological changes in cultured populations of four bacterial species, *Bacillus subtilis, Lactiplantibacillus plantarum, Pediococcus acidilactici* and *Escherichia coli.* Morphologies in relation to growth stage and stress responses, cell integrity and metabolic activity were analysed. Additionally, we were able to identify and morphologically classify dormant cell phenotypes such as VBNC cells and monitor the resuscitation of persister cells in *Escherichia coli* following antibiotic treatment. We therefore demonstrate that IFC, with its high-throughput data collection and image capture capabilities, provides a platform by which a detailed understanding of changes in bacterial phenotypes and their physiological implications may be accurately monitored and quantified, leading to a better understanding of the role of phenotypic heterogeneity in the dynamic microbiome.

## Introduction

Bacteria exhibit a wide range of cell morphologies including cocci (spheres), bacilli (rods) and spirochaetes (spirals), characteristics that, prior to DNA sequencing, were used to classify species (Cabeen and Jacobs-Wagner, 2005). Morphological diversity among bacteria potentially reflects species’ evolution as they adapted to survive in varied environments (Young, 2006). Although bacteria typically maintain their characteristic shape through multiple generations, they can modify their morphology throughout their life cycle, sometimes in response to environmental factors (van Teeseling et al., 2017). A single bacterial species can therefore exhibit various physiological and physical phenotypes such as changes in size and shape, resulting in high levels of heterogeneity within a population (Kundu et al., 2020).

Morphological plasticity in bacteria is often exhibited during different growth states. For example, some rod-shaped bacteria may exhibit exaggerated cell elongation during periods of rapid growth (Chang and Huang, 2014) or transition to small coccoids when growth is static or slow (van Teeseling et al., 2017). Environmental stresses, including the availability of nutrients (Westfall and Levin, 2018), oxygen (Murashko and Lin-Chao, 2017), and pH changes (Ingham et al., 2008) may also result in bacteria adapting their shape in response to these environmental changes. Examples of such adaptation include the production of spores in *Bacillus subtilis* in response to starvation (McKenney et al., 2013), the generation of filamentous cells in *Lactoplantibacillus plantarum* in response to acidification (Ingham et al., 2008) and exposure to antibiotics (Maki et al., 2000) or the transition to a dormant “viable but non-culturable” (VBNC) state in response to potentially lethal changes in the environment (Dewachter et al., 2019; Wagley et al., 2021). These dormant states in bacteria are often accompanied by a shift in morphology as their metabolic state changes. Similarly, exposure to antibiotics can induce a drastic change in physiology that is accompanied by a change in morphology to generate so-called “persister cells”. Persister cells are not genetically resistant to antibiotics but can tolerate lethal levels of antibiotics. Since persister cells can revive when antibiotic stress is removed, they are critical in infection relapses (Fisher et al., 2017). Cell shape has also been shown to influence a number of other processes that are vital for bacterial life-strategies including biofilm formation (Smith et al., 2017), motility (Portela et al., 2019), predation (Justice et al., 2008) and can aid bacteria in evading detection by immune systems (Baranov et al., 2021), thus influencing bacteria pathogenicity (van Teeseling et al., 2017).

Despite the importance of size and shape as a physiological property, acquiring qualitative and quantitative data about bacterial cell morphology is challenging and the inherent heterogeneity of phenotypes exhibited by different species is therefore often overlooked. High-throughput morphological data for bacterial populations is unachievable with the standard techniques used to monitor cell growth (optical density and colony forming unit counts), and is constrained by time consuming microscope techniques (Uzoechi and Abu-Lail, 2020). Whilst conventional flow cytometry provides high-throughput data acquisition, cell morphology detection is restricted to forward and side scatter properties (Narayana et al., 2020). Microfluidic technologies that provide high-resolution cell imaging are typically designed to monitor growth from isolated single cells and thereby restricted to the analysis of relatively small cell populations (Rusconi et al., 2014).

In this investigation we used Imaging Flow Cytometry to monitor the changing morphological characteristics of bacteria over time and in response to certain environmental challenges. *Bacillus subtilis* was selected as the model Gram-positive, rod-shape species, and key phenotypes during exponential and stationary growth phases were characterised. We also tracked the morphology, viability and metabolic activity of two lactic acid bacteria of differing shapes, *Lactiplantibacillus plantarum* (rod-shaped) *and Pediococcus acidilactici* (coccoid-shaped), as they grew and responded to an increasingly acidic environment. Finally, to demonstrate how IFC can be used to monitor stress-induced characteristics, we analysed the phenotypic responses of *Escherichia coli* (DH5α), the model Gram-negative (rod-shaped) bacterium, when exposed to short-term antibiotic and long-term antibiotic treatments to detect VBNC and persister cell sub-populations.

Our work demonstrates the application of IFC for detailed detection and quantification of bacterial phenotypes to monitor rapidly growing cells and the physiological adaptations to stress. We also identified dormant cell phenotypes in this high-throughput manner that occur in response to antibiotic stress. The high-throughput and high-resolution, cell-by-cell, phenotypic monitoring that is enabled by IFC has the potential to revolutionise a range of environmental, industrial and public health-related microbiological applications including immunology and understanding host-pathogen interactions.

## Materials and Methods

### Bacterial Strains, Media and Culture Conditions

*Bacillus subtilis* WB800N purchased from Mo Bi Tech was cultured under atmospheric oxygen conditions at 35°C for 24 h, continuously shaking at 220 rpm. Culture volumes were 50 ml LB (Lysogeny Broth, aka. Luria-Bertani) (Melford L24400-5000.0) in 250 ml Erlenmeyer flasks.

*Lactiplantibacillus plantarum* WCFS1 purchased from ATCC (BAA-793)**(formally *Lactobacillus plantarum*) and *Pediococcus acidilactici *(DSM 20238) were grown anaerobically in 10% CO_2, _10% H_2_, 80% N_2_ (BOC anaerobic growth mixture) at 35 °C for 48 h. Culture volumes were 6 ml MRS media (de Man, Rogosa, Sharpe) in 50 ml centrifuge tubes (Greiner). Overnight cultures (5 ml) were prepared and used to inoculate 6 ml to a final optical density at 600 nm (OD_600_) of 0.1 (Tecan Infinite Mplex). MRS recipe: 10g l^-1^ Casein peptone tryptic digest (tryptone), 10g l^-1^ Meat extract, 5 g l^-1^ Yeast extract, 1 ml l^-1^ Tween 80, 2% w/v Glucose, 11.5 mM K_2_HPO_4_, 61 mM Sodium acetate, 8.8 mM Ammonium citrate, 0.8 mM Magnesium sulphate and 0.3 mM Manganese sulphate.

*Escherichia coli* ****DH5α was grown under atmospheric oxygen, at 37°C, continuously shaking at 220 rpm. Culture volumes were 50 ml LB Broth in 250 ml Erlenmeyer flasks. 10 ml overnight cultures prepared in 50 ml centrifuge tubes were used to inoculate LB media to a final OD_600_ of 0.1 (Tecan infinite Mplex). Samples were taken each hour for the duration of the experiment. A total of 100 µl of culture was removed for IFC analysis and 50 µl of diluted culture was plated onto LB agar for colony forming unit (CFU) enumeration.

All experiments were performed with at least 3 biological replicates (*n* ≥ 3).

### Determination of Minimum Inhibitory Ampicillin Concentrations (*Escherichia coli)*

To determine the minimum inhibitory concentration of ampicillin, a dose response curve was produced for *Escherichia coli* ****DH5α (**Supplementary Figure 1**). Overnight cultures of *E. coli* in LB were used to inoculate 50 ml LB with ampicillin in 250 ml Erlenmeyer flasks to a final OD_600_ of 0.1 (Tecan infinite Mplex). Concentrations of ampicillin used were 100, 50, 37.5, 25, 12.5, 6.25, 3.13, 0 µg ml^-1^. OD_600_ was then measured every hour for 24 h.

### Antibiotic Treatment and Resuscitation (*Escherichia coli*)

After 2 h incubation in LB broth, ampicillin was added to each flask culture to a final concentration of 100 µg ml^-1^. Cultures were then incubated for a further 6 h (short-term treatment) and 16 h (long-term treatment). After incubation in the presence of ampicillin, 10 ml of culture was removed from each flask and spun at 8000 rpm for 5 mins. Supernatant was removed and the pellet resuspended in fresh LB media. This resuspension was used to inoculate 50 ml LB media which was incubated for a further 4 h (short-term treatment) or 8 h (long-term treatment) resuscitation period.

### Preparation of Samples for Imaging Flow Cytometry

For *B. subtilis* WB800N, 200 µl of sample was diluted to a final OD_600_ of 0.2 (Tecan infinite Mplex) at each timepoint prior to IFC analysis. Samples were analysed at 0 h, 2 h, 4 h, 6 h to represent exponential growth and again at 8 h and 20 h to represent stationary phase.

For *L. plantarum* WCFS1, *P. acidilactici* and *E. coli*, 100 µl samples were stained using the Invitogen Baclight Redox Sensor Green (RSG) Vitality kit (Thermo Fischer Scientific catalogue number: B34954). Each 100 µl sample had a final RSG concentration of 1µM and a final propidium iodide (PI) concentration of 1.5 µM. Samples were protected from light and incubated at room temperature for 15 minutes before analysis. Samples were washed in phosphate buffered saline (PBS) and diluted or concentrated as required for optimal IFC acquisition and acquired at selected timepoints.

### Imaging Flow Cytometry Data Acquisition

Data acquisition was performed using a fully calibrated (ASSIST tool) ImageStream X MkII (ISX, Luminex Corp, Seattle, USA) configured with a single camera and 405, 488, 561, 642 and 785 nm excitation lasers, brightfield (BF) illumination, multi-magnification capabilities (20X, 40X and 60X) and a six-channel detection system.

A hydrodynamically focused stream allowed each object within a sample to be analysed individually. Interrogated by a suite of lasers and BF illumination, emitted light was detected on a series of channels (Ch01, Ch02, Ch03, Ch04, Ch05 and Ch06), each relating to a specific bandwidth. Brightfield (BF) images were collected on Ch04 (BF, 610/30 nm) and side scatter (SSC) on Ch06 (SSC, 762/35 nm). RSG signals were detected on Ch02 (533/55 nm), and PI on Ch05 (702/85 nm).

For maximum resolution and high sensitivity, fluidics were set at low speed. Magnification was set at 60X objective (0.3 µm^2^ pixel resolution). Brightfield illumination and excitation lasers 488 nm (at 1.00 mW), 561 nm (at 100 mW) and 785 nm (at 2.50 mW) were applied to determine RSG, PI and SSC signals, respectively. Images were collected at a rate of up to 1,000 cells s^-1^ until 20,000 objects of interest were acquired (10,000 for *B. subtilis* experiment). A broad gating strategy (Intensity of Ch02 < 1,000 and Intensity Ch06 > 40,000) excluded the majority of speed beads (microspheres used to maintain camera focus during analysis). A compensation matrix was applied to adjust for spectral overlap between channels, calculated from data acquired excluding BF and SSC excitation. Blank media controls were acquired to identify any potential contaminants.

### Imaging Flow Cytometry Data Analysis

Analysis of IFC data was performed using IDEAS^®^ software (Version 6.2, EMD Millipore, Seattle). Bespoke ‘masks’, regions superimposed over channel images used to calculate feature values, were defined to enable accurate quantitative morphological data.

To ensure high-quality data sets, background objects, out of focus images and image captures containing multiple objects were identified and excluded *via* sequential gating of Gradient RMS, Contrast and SSC intensity of BF thresholds (**Supplementary Figure 2**). Propidium iodide signals were normalised by cell size, since fluorescence intensity increases with object size (**Supplementary Figure 3**), allowing an accurate determination of cells with damaged cell membranes. Positive RSG signal intensities were identified by initially determining background Ch02 intensities using negative controls (**Supplementary Figure 4**).

Morphological features and fluorescence intensity data was extracted using statistical analyses within the IDEAS software. Graphs were constructed, and further statistical analyses were performed using Prism (version 9.0.0).

### Statistical Analyses

Results are expressed as mean, with standard error of the mean (SEM), unless stated. One-way ANOVA (analysis of variance) was applied to identify significant differences in population dynamics at varying time points. Anderson-Darling normality and lognormality tests were applied to assess data distribution. Data were either log-normally distributed, and therefore transformed or, where stated, Spearman’s Rank (non-parametric) correlations were applied to determine significant relationships. For statistical tests, *p-*values < 0.05 were considered statistically significant.

### CFU Enumeration, pH and OD_600 nm_ Measurements

Colony-forming units (CFU) were enumerated after serial dilution of cultures onto LB agar. The pH was monitored using an Orion 9110DJWP double junction pH electrode. Bacterial growth was monitored *via* optical density at 600 nm (OD_600_) using a spectrophotometer (Tecan Infinite Mplex).

## Results

### *Bacillus subtilis* Growth Phenotypes

**Figure 1:**
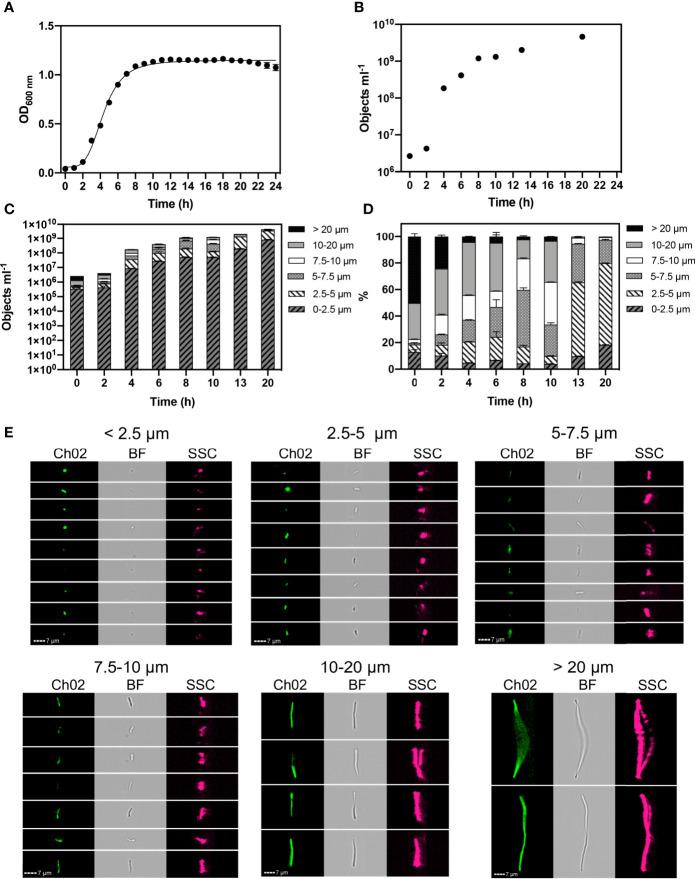
Monitored phenotypes of *Bacillus subtilis* WB800N during 24 h growth. Hourly optical density (OD_600_) measurements were performed **(A)**. Imaging Flow Cytometry was used to determine: cell concentration (objects ml^-1^) **(B)**. Concentrations of different cell morphologies (as indicated by cell length) **(C)** and their relative proportions **(D)** are presented at selected time points. Exemplar cell images of autofluorescence (Ch02: 533/55 nm), brightfield (BF: 610/30 nm) and side scatter (SSC: 762/35 nm) within selected cell length intervals are shown **(E)**. *n= 3*; error bars represent standard error of the mean.

*B. subtilis* is known to exhibit variations in cell length whilst maintaining a uniform cell width (Cabeen and Jacobs-Wagner, 2005). Therefore, we monitored the length of cells to classify changes in the morphology of *B. subtilis* over 20 h of growth using IFC (**Figure 1**). Optical density measurements were performed hourly over a 24 h period. *B. subtilis* exhibited exponential growth from 2-8 h, prior to entering stationary phase (**Figure 1A**). IFC was applied to quantify and morphologically characterise cell changes. Bacterial cell counts (**Figure 1B**) correspond with OD_600_ measurements (**Figure 1A**).

We found that *B. subtilis* cells can achieve lengths exceeding 20 µm. Concentrations of cell size fractions (**Figure 1C**) and their proportions (**Figure 1D**) were measured at selected timepoints to reflect log and stationary growth phases. Size classifications of 2.5 µm intervals were applied to determine changes in cell length at high-resolution. We classified individual, small cells as < 2.5 µm, and tracked cell elongation at consistent intervals of 2.5 µm up to 10 µm. Broader size intervals were used to include highly elongated chains cells: 10-20 µm and >20 µm (**Figure 1E**).

During the first 2 h of growth, *B. subtilis* cell concentrations were relatively low (4.3 x10^6^), however they were dominated by elongated cells, with ~77% of the population exceeding 10 µm in length at 0 h and ~60% at 2 h (**Figure 1D**). A reduction in cell length gradually occurred throughout exponential growth (2- 8 h), reflecting a rapid division of cells. This was confirmed in our experiments where we observed ~15% of the cells were > 10 µm in size at stationary phase (8 h). Cell length continued to reduce throughout stationary phase. At 20 h ~2% of cells are > 7.5 µm, with the majority of cells (~62%) exhibiting a cell length of 2.5 – 5 µm. This distinct shift to a small cell state, as nutrients becomes limited, reflects a change in the physiological properties of cells as they cease growing and activate a survival response.

### Lactic Acid Bacteria Growth Phenotypes

Lactic acid bacteria such as *L. plantarum* (rod shaped) and *P. acidilactici* (coccoid shaped), are non-spore forming, facultative anaerobes that can ferment in the presence and absence of oxygen and tolerate a range of temperature and pH environments. *P. acidilactici* is homofermentative (Bansal et al., 2019), whereas *L. plantarum* WCFS1 can switch between homofermentative and heterofermentative metabolism whereby ethanol, acetic acid and carbon dioxide are also produced (Botta et al., 2017). The production of lactic acid results in the bacteria progressively acidifying their environment as they grow, thereby introducing a self-imposed stress on the microbiome.

*L. plantarum* WCFS1 and *P. acidilactici* were grown anaerobically over 48 h whilst monitoring OD_600_ and pH. Changes in cell phenotypes were classified and quantified using IFC at selected time points. We applied propidium iodide (PI), a DNA binding stain that penetrates damaged cell membranes, as an indicator of cell viability. Cells with compromised membranes therefore yielded a red fluorescence signal (Rosenberg et al., 2019). Redox sensor green (RSG) was also applied as a fluorescence marker for metabolic activity which yielded a green fluorescence signal.

RSG penetrates cells and, in the presence of reductase enzymes, fluoresces green, indicating metabolic activity that of the electron transport chain (ETC) at the single‐cell level (Konopka et al., 2011). *L. plantarum* and *P. acidilactici* ferment sugars to lactic acid *via* the glycolysis pathway, which does not require ETC activation. Genomic assessments have revealed that *L. plantarum* exhibits a ‘rudimentary ETC’, with the potential of a nitrate reduction system (Kleerebezem et al., 2003; Botta et al., 2017). In the presence of heme (present in MRS as a component of meat extract) *L. plantarum* can perform anaerobic respiration (Zotta et al., 2014), in which the ETC is activated (Brooijmans et al., 2009), and nitrate is used as a final electron receptor. Therefore, we used RSG as an indicator of the NAD+ to NADH redox reaction that drives metabolic activity during anaerobic fermentation and, potentially anaerobic respiration in the *L. plantarum* cultures.

#### *Lactiplantibacillus plantarum* Growth Phenotypes

**Figure 2:**
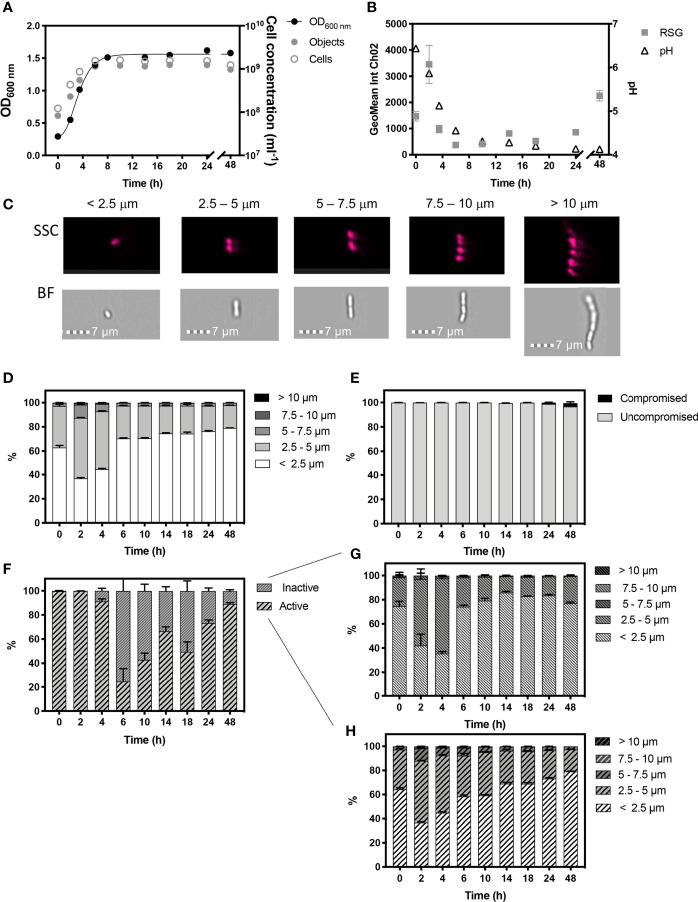
Monitored phenotypes of *Lactiplantibacillus plantarum* WCFS1 during 48 h growth. Measurements of Optical density (OD_600_) are plotted against IFC determined cell concentrations displayed as objects (ml^-1^) and cells (ml^-1^) **(A)**. Cells ml^-1^ was calculated *via* normalization of object concentration by cell size and used as a proxy for biomass. The geometric mean of redox sensor green (RSG), indicative of metabolism, was determined from Channel 2 (Ch02: 533/55 nm) intensity and is plotted alongside media pH **(B)**. Cell phenotypes were characterised by cell length, determined by brightfield (BF) and side scatter (SSC) properties **(C)**. Proportions of cell length during growth are shown **(D)**. The proportion of cells with compromised and uncompromised cell membranes, was tracked using propidium iodine and determined by red fluorescence intensity (Ch05: 702/85 nm), normalised for cell area **(E)**. Proportions of metabolically ‘active’ and ‘inactive’ cells are presented **(F)** and cell lengths of ‘inactive’ **(G)** and ‘active’ **(H)** cells within the uncompromised population are shown*. n =5*; error bars represent standard error of the mean.

In these experiments, OD_600_ and cell concentration determined by IFC showed *L. plantarum* (**Figure 2**) grew exponentially until 8 h when stationary phase was reached (**Figure 2A**). Cell concentrations are presented as the number of individual objects ml^-1^ detected by IFC and the total number of cells ml^-1^, whereby object number has been normalised for cell length (**Figure 2A**). The cell length (determined from BF images) of *L. plantarum* was classified into 2.5 µm intervals, since SSC and BF data revealed that this size range reflected increases from single cells (< 2.5 µm), pairs (2.5-5 µm), chains of ≈3 cells (5-7.5 µm), ≈4 cells (7.5-10 µm) and cells > 10 µm in length (**Figure 2C**).

Cell morphology changed dynamically throughout growth (**Figure 2D**). Exponential growth was characterised by a relatively heterogenous cell length, with approximately half the population at 2 h and 4 h being 2.5 -5 µm in length. A total of ~13% and ~7% of cells in the total population were long chains that exceeded 5 µm in length at 2 h and 4 h, respectively. This size distribution likely reflects a characteristic of rapidly growing cells, as seen in *B. subtilis* (**Figure 1**).

Stationary phase commenced from 8 h, and is likely to be initiated by a combination of glucose deprivation and acidification. During this stage *L. plantarum* exhibited a more homogenous, size distribution with 79% of the cell population being < 2.5 µm in length.

*L. plantarum* demonstrated high cell viability, with over 99.80% of the population showing uncompromised cell membranes, as determined by the discrimination of healthy, viable cells from damaged, compromised cells exhibiting red fluorescence signals indicative of PI staining over the 8 h period. However, red fluorescence increased in cells to 1.14% by 24 h and to 2.69% by 48 h, indicating an increase in cells with compromised integrities at these later time-points (**Figure 2E**). During the first 2 h of growth, 99.76% of the cell population showed a peak in mean RSG intensity indicating cells in the population were metabolically active (**Figure 2F**), which is reflected by the onset of rapid cell growth, and was corroborated by a rapid decline in pH from 0 h - 4 h (**Figure 2B**) due to the production of lactate. However, by 6 h, the proportion of cells in the population with metabolic activity was reduced to 24.50%, after which we observed a steady increase throughout stationary phase to 89.02% at 48 h, and which we ascribe to a metabolic response to stress. The cell morphology of metabolically ‘active’ and ‘inactive’ cells was also characterised (**Figures 2G, H**) and shows a similar distribution of morphological phenotypes.

#### *Pediococcus acidilactici* Growth Phenotypes

*P. acidilactici* is a lactic acid bacteria that is morphologically distinct from *L. plantarum* and presents as uniform cocci that do not form chains but are mainly composed of pairs and tetrads, that remain attached together following division along a single plane, in two perpendicular directions (Zapun et al., 2008; Bintsis, 2018).

**Figure 3:**
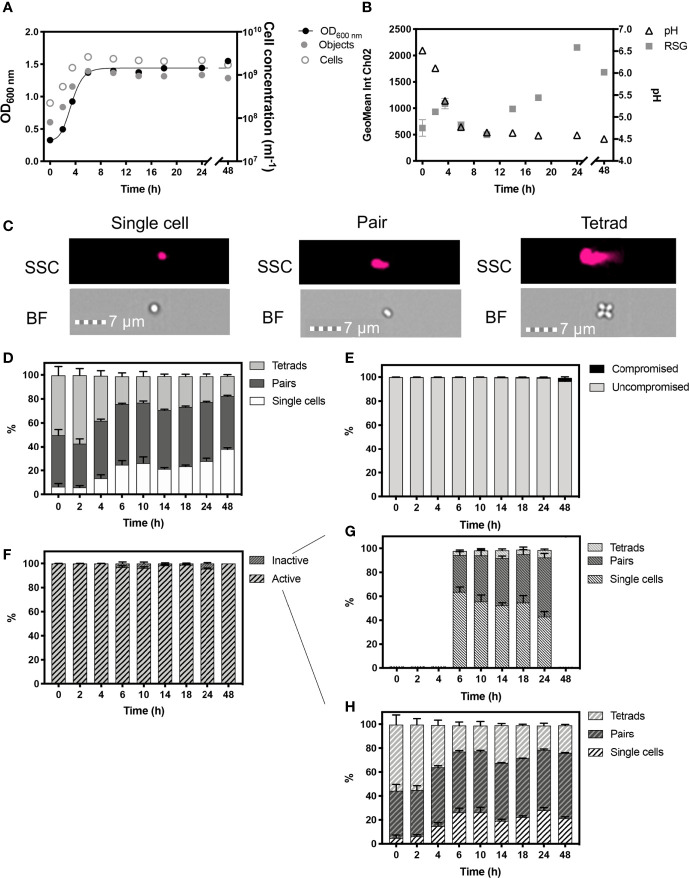
Monitored phenotypes of *Pediococcus acidilactici* during 48 h growth. Measurements of optical density (OD_600_) are plotted against IFC determined cell concentrations displayed as objects (ml^-1^) and cells (ml^-1^) **(A)**. Cells ml^-1^ was calculated *via* normalization of object concentration by cell type and used as a proxy for biomass. Geometric mean of redox sensor green (RSG), indicative of metabolism, was determined from Channel 2 (Ch02: 533/55 nm) intensity, and is plotted alongside media pH **(B)**. Cell phenotypes were characterised by cell length, determined by brightfield (BF) and side scatter (SSC) properties **(C)**. Proportions of cell length during growth are shown **(D)**. The proportion of cells with compromised and uncompromised cell membranes, was tracked using propidium iodine and determined by red fluorescence intensity (Ch05: 702/85 nm), normalised for cell area **(E)**. Proportions of metabolically ‘active’ and ‘inactive’ cells are presented **(F)**. Cell lengths of ‘inactive’ **(G)** and ‘active’ **(H)**: cells within the uncompromised population are shown. *n = 5*; error bars represent standard error of the mean.

Unlike *L. plantarum*, and *B. subtilis*, whose rod-shaped cells were aligned along their longest plane during IFC acquisition, the spherical cells of *P. acidilactici* did not exhibit a uniform orientation during analysis. Therefore, BF images cannot be used alone to account for any morphological differences in this species. In this study we found that SSC was more accurate in detecting the presence of multiple cells, and was therefore used to classify single cells, pairs, and tetrads in these experiments (**Figure 3C**).

Optical density measurements showed exponential growth in *P. acidilactici* occurred until ≈6 h (**Figure 3A**). Cell concentrations are presented as objects ml^-1^ and cells ml^-1^, the latter normalised for cell phenotypes, with single cells representing 1 cell, pairs representing 2 cells and tetrads representing 4 cells (**Figure 3C**). The changing proportions of these cell phenotypes were tracked over 48 h of growth (**Figure 3D**). *P. acidilactici* exhibited dynamic cell attachment during growth, with the highest proportion of tetrads (~57%) and the lowest proportion of single cells (~5.80%) present in the population at 2 h. By the start of stationary phase at 6 h, ~25% of the population was comprised of single cells and there is a corresponding reduction in the number of tetrads observed (23%). The proportion of single cells in the population continued to increase throughout stationary phase and, by 48 h, the proportion of single cells was ~38% of the total population. The proportion of pairs of cells, however, remained relatively constant throughout stationary phase, comprising ~50% of the population. P*. acidilactici* progressively acidified the growth media to pH 4.5 at 48 h from an initial 6.5 at 0 h (**Figure 3B**) however, the vast majority of cells (> 99.2%), remained viable as indicated by a lack of membrane damage, determined by PI staining. A slight reduction in cells with intact cell membranes occurred at 48 h (96.50%; **Figure 3E**).

The majority of cells exhibited a positive RSG signal, with > 99.8% of cells showing metabolic activity from 0 – 4 h, with an initial peak in RSG geomean at 4 h that is likely to reflect rapid metabolism during exponential phase (**Figure 3F**). Approximately 4% of cells are ‘inactive’ at 6, 10 and 24 h as the fermentation of glucose to lactic acid slows, as demonstrated by stabilised pH levels. The ‘inactive’ cell population is characterised by the relatively higher proportions of single cells (**Figures 3G, H**). Maximum values in the geometric mean intensity of RSG occurred at 24 h, potentially indicating a metabolic switch, as a stress response, which coincides with the increase in the relative number of single cells.

### Characterisation of Antibiotic-Induced Phenotypes in *Escherichia coli*

#### Discrimination of Persister Group Cells From Lysed Cells During Antibiotic Treatment in *E. coli*

**Figure 4:**
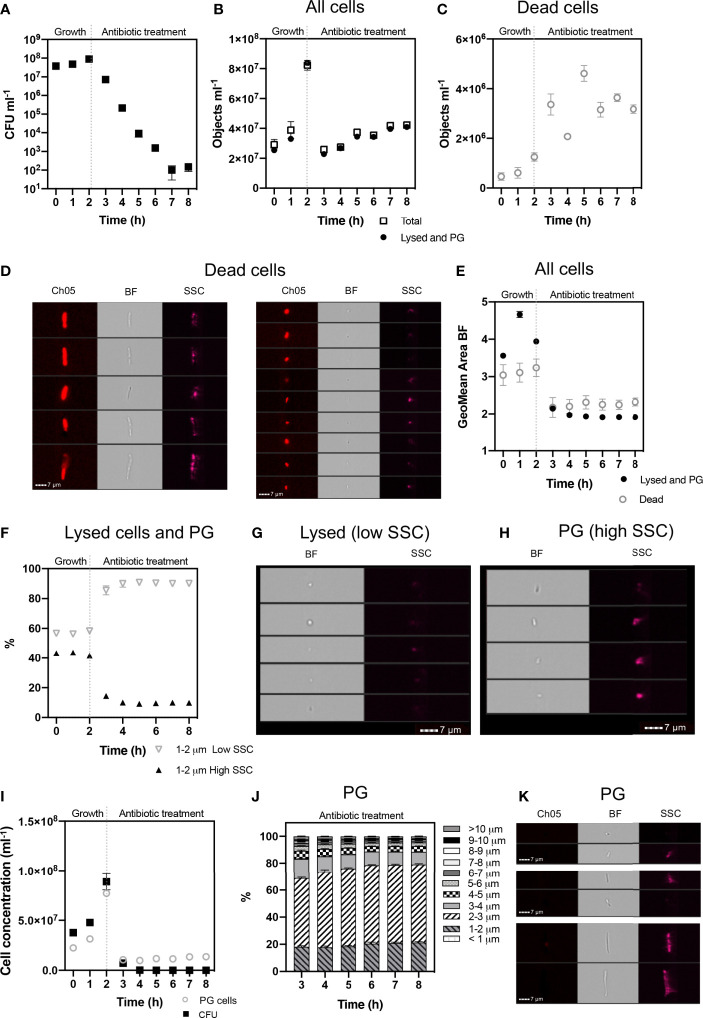
Cell phenotypes of E. coli during 2 h exponential growth and 6 h antibiotic treatment. The growth of viable cells was monitored via colony forming unit (CFU) counts **(A)**. IFC was used to detect cell concentrations (objects ml-1) of the ‘Total’ population and ‘Lysed and Persister Group’ (PG) cells (i.e., persister cells and VBNC) **(B)**. Concentrations of ‘Dead’ cells **(C)** were identified based on their high PI signal (Ch05: 702/85 nm) and pitted morphology **(D)**. Dead cells were excluded from ‘Total’ cells, to determine ‘Lysed and PG’ cells. The geometric mean area of cells demonstrates a decrease in cell size following antibiotic treatment **(E)**. Lysed cells were determined as having low side scatter (SSC) intensity and increased on the application of antibiotic **(F)**. Brightfield (BF) and SSC images demonstrate the low SSC cells (Lysed) have a compromised cell structure, consisting of small spherical particulate **(G)**, indicative of lysed debris. In comparison, high SSC (PG) cells demonstrate a rod-shaped structure, highlighting cell integrity **(H)**. The gating strategy applied for isolating lysed cells from PG cells, is supported by statistically significant relationship between CFU counts and PG cell concentrations **(I)** (Spearman’s Rank: p < 0.05). Size proportions of the PG cells are shown in 1 µm intervals **(J)** and exemplar cells from the PG population at 8 h are shown **(K)**. n = 4; error bars represent standard error of the mean.

**Figure 5:**
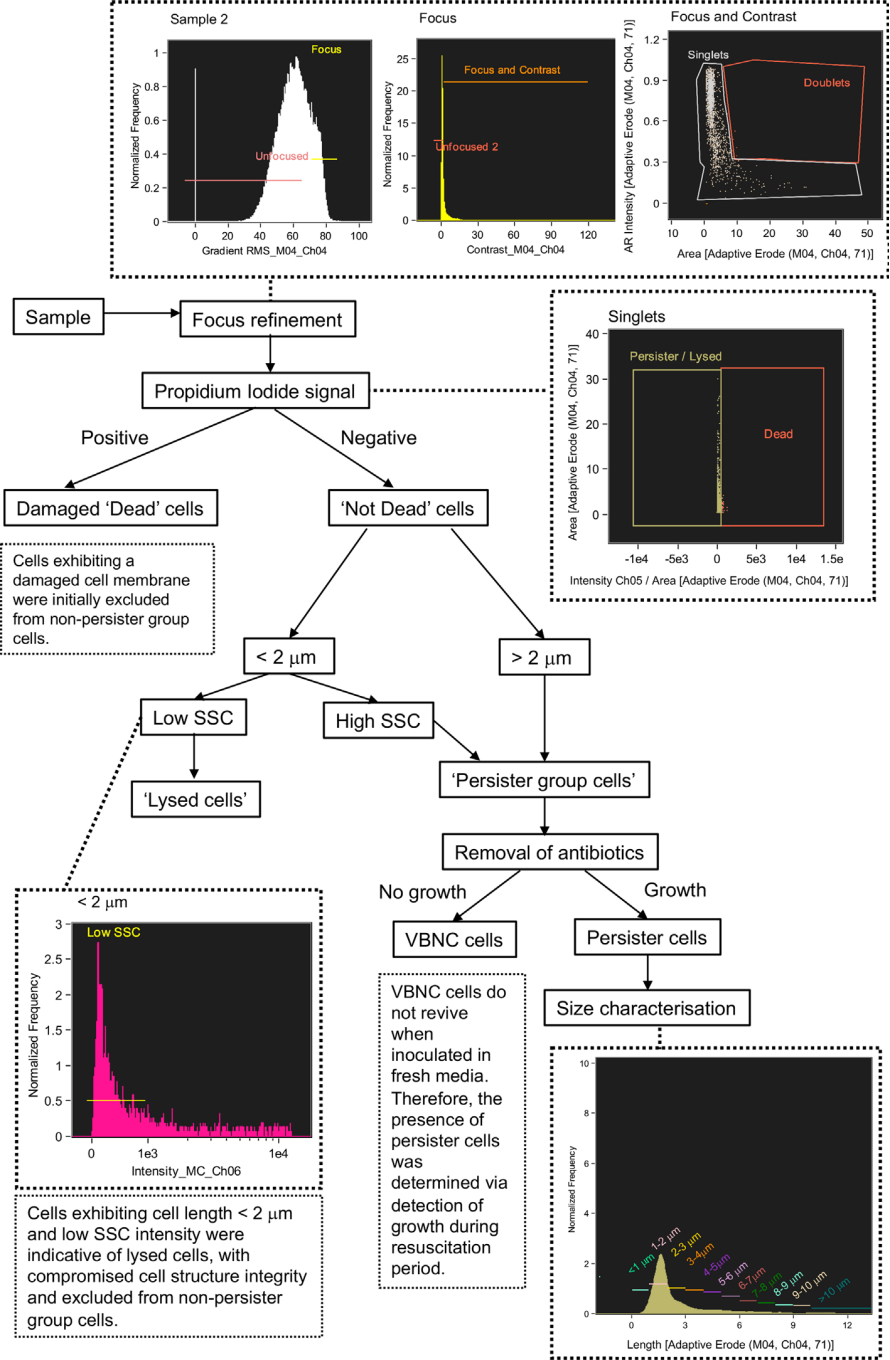
Flow diagram of steps taken to discriminate between lysed cells, damaged cells, viable but non culturable cells and persister cells using Imaging Flow Cytometry.

IFC was used to analyse cell phenotypes in *E. coli* cultures grown to mid-log phase (2 h) and then exposed to a short (6 h) ampicillin treatment. This treatment was sufficient to lyse antibiotic susceptible cells (*i.e.* non-persister group) as shown by the bi-phasic kill curve from the CFU counts (**Figure 4A**) and total inhibitory growth at 6 h. Damaged cells, determined as dead and lysed cells arising from the antibiotic treatment, were initially discriminated and the remaining undamaged cells were monitored to detect the survival phenotypes: viable but non culturable (VBNC) cells and persister cells (PC) (**Figures 4**, **5**). Cells were characterised by their length, at intervals of 1 µm to detect subtle morphologies that may be indicative of distinct phenotypes.

A defining characteristic of VBNC and persister cells is that the cell membrane is not damaged or compromised and thus the PI stain is excluded from the cytoplasm and the cells remain non-fluorescent (Orman and Brynildsen, 2013). This feature allowed an initial discrimination of ‘dead cells’, which had a strong red fluorescence due to the absorbed PI. Brightfield and SSC images revealed that dead cells exhibited a pitted appearance, highlighting cell damage from the antibiotic treatment (**Figure 4D**). The concentration of dead cells initially increased with the application of ampicillin at 3 h and declined at 6 h as the amount of lysed cell debris increased (**Figure 4C**).

Lysed cell debris was distinguished from persister group cells (**Figure 4B**) based on distinctive size and SSC properties. In previous studies, the application of ampicillin to *E. coli* resulted in a shift to relatively small cell particulate as cells become lysed (Mohiuddin et al., 2020). We demonstrated a distinct shift in mean cell area from 3.941 µm^2^ during mid log (2 h) when the cells were growing and elongating, to 2.146 µm^2^ after 1 h exposure to ampicillin (culture t = 3 h) and 1.906 µm^2^ after 6 h exposure (culture t = 8 h) (**Figure 4E**). Analysis of cells < 2 µm in length revealed an increased proportion of cells with a low SSC intensity from ~58% at 2 h to ~87% at 3 h, *i.e.* after 1 h exposure to ampicillin, and then to 90.5% at 8 h (*i.e.* after 6 h exposure to ampicillin) (**Figure 4F**). The low SSC obtained from these objects indicated a lack of cell structure integrity and cell lysis.

Cells that were < 2 µm and with low SSC were therefore classified as lysed cell debris. Conversely, cells < 2 µm with high SSC intensities were classified as belonging to the Persister Group (PG) comprising VBNC and persister cells. This distinction was supported by visual inspection of the brightfield images for these cells, which show the high SSC population consisted of the characteristic rod-shaped cells of healthy *E. coli* (**Figure 4H**), in contrast to the more amorphous particulate of lysed cell debris (**Figure 4G**).

The strategy used here to identify and exclude lysed cells from the PG (**Figure 5**) was further supported by the close parallels between the PG concentrations as determined by IFC and the CFU counts (**Figure 4I**), which demonstrated a statistically significant relationship (R = 0.560 *p* = 0.05 Spearman’s Rank or lognormally transformed Pearson’s R = 0.768 *p* = 0.002). During the initial growth phase (0 - 2 h), cells classified as PG were indicative of all cells with uncompromised cell membranes (as determined by PI), excluding cells < 2 µm with low SSC. During antibiotic treatment, the PG group was indicative of the true dormant population with a concentration of 1.355 x 10^7^ objects ml^-1^ at 8 h. This PG group was characterized by a range of cell lengths up to >10 µm, however the majority of cells (≈ 80%) measured less than 4 µm in length, and ≈ 50% of cells were 2-3 µm in length (**Figures 4J, K**).

#### Distinction of Persister Cells From VBNC Cells

Persister cells are a rare dormant phenotype within a bacterial population, typically comprising < 1% of cells (Bamford et al., 2017; Dewachter et al., 2019). Persister cells tolerate otherwise lethal concentrations of antibiotics and have the ability to regrow when the antibiotic stress is removed (Mohiuddin et al., 2020). The accurate detection of persister cells following antibiotic treatment is limited due to their scarcity and requires their isolation from lysed cell debris and VBNC cells (Orman and Brynildsen, 2013; Mohiuddin et al., 2020). In this investigation, persister cells were identified by their regrowth in fresh LB media following a long-term (16 h) ampicillin treatment. Persister cells were discriminated from dead and lysed cells using criteria determined during the previous short-term antibiotic treatment (**Figure 4**), and from VBNC cells which do not grow on standard media (Orman et al., 2016; Mohiuddin et al., 2020).

**Figure 6:**
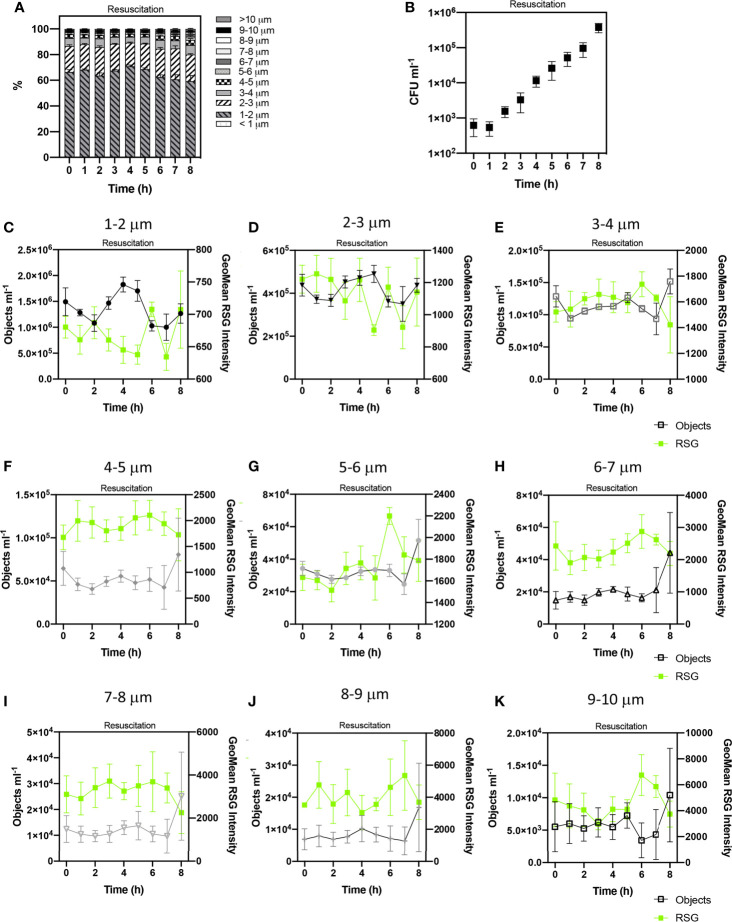
Tracking the revival of *E. coli* persister cells after antibiotic exposure. Proportions of cell sizes within the persister group (PG) **(A)**. The growth of viable cells was monitored via colony forming unit (CFU) counts **(B)**. Concentrations of cell sizes (at 1 µm cell length intervals) and Geometric mean intensity of RSG for these size intervals over 8 h of resuscitation **(C–K)**. Changes in these concentrations reflect cell growth and therefore the determination of persister cells. n = 5; error bars represent standard error of the mean.

Cells treated with ampicillin were washed and inoculated in fresh LB to revive any persister cells. Regrowth was monitored over 8 h by CFU counts (**Figure 6B**) and IFC analysis was used to monitor changes in cell phenotypes. We observed and recorded increases in cell concentration numbers and elongation which are indicative of cell growth (**Figure 6A**). The regrowth of *E. coli* persister cells has also been characterised by an activation of the ETC and ATP production (Wilmaerts et al., 2019) and therefore, metabolic activity was also analysed using the RSG stain (**Figures 6C–K**).

CFU counts showed that persister cells started to regrow from 4 – 8 h (**Figure 6B**) which corresponds with IFC data that demonstrated changes to cell size, indicative of cell revival at this time. Statistical analyses (ANOVA) indicated no significant variation in the concentration of cell phenotypes, suggesting no detectable growth during the first 4 h of resuscitation, as observed following the short-term treatment (**Supplementary Figure 5**) (*p* > 0.05 in 16 h ampicillin treatment and *p* > 0.05 in 6 h ampicillin treatment). This extended lag phase characterised by ≈ 4 h minimal growth, and relatively diminished CFU concentrations over 8 h and contrasting to the initial 2 h growth phase in media containing no antibiotic (**Supplementary Figure 6**) has also been reported for *E. coli* (Balaban et al., 2004) and potentially reflects the requirement for persister cells to repair DNA damage and active division (Dewachter et al., 2019). The morphology of cells at the start of resuscitation were statistically similar following both the long-term (16 h) and short-term (6 h) ampicillin treatments (unpaired t test p> 0.05 and p> 0.05 respectively). At 0 h the PG is comprised of ~90% cells < 3 µm in length with the remaining ~10% comprised of cells from 3 µm to 10 µm in length.

Changes in cell concentrations were detected after 4 h resuscitation, with statistically significant variation over 8 h resuscitation (determined by ANOVA, p= < 0.0001) and significant variation at 4 h, 5 h and 7 h timepoints (as revealed by Tukey- Kraymer test p < 0.05). These changes in the dynamics of cell concentrations from 4-8 h was indicative of cell growth.

We detected an initial increase in cells measuring < 3 µm in length at 4 h and 5 h. Temporal trends in the concentrations of cells < 1-2 µm and 2-3 µm in length during the 8 h revival period were statistically related to each other (Spearman’s R=0.95 p=< 0.0001), whilst being statistically independent from cell size classifications > 3 µm, potentially highlighting a persister cell group made up of cells < 3 µm.

At 8 h, we observed notable increases in all cell lengths exceeding 3 µm, potentially reflecting the elongation of the initial < 3 µm persister cells to longer cells (> 3 µm). Significant statistical relationships were detected between cells 3-4, 4-5, 5-6 and 7-8 µm in length (Spearman R values 0.83 – 0.88, p values < 0.01) (**Supplementary Figure 7**). Cells 7-8 µm in length were statistically related to cells 8-9 µm (Spearman’s R=0.82, p=0.011). These relationships highlight corresponding trends in the growth of these size classifications during resuscitation. This further supports the initial growth of cells < 3 µm at 4-5 h and subsequent elongation of these cells from 5-8 h.

Significant correlations between cell growth and RSG intensities for the cell length intervals were not detected.

## Discussion

### The Application of IFC for Analysis of Different Cell Changes During Growth Phases

The widely studied *B. subtilis*, has been shown to reduce its cell size as a defence mechanisms to protect its DNA, including nucleoid condensation (Exley et al., 2001), ribosome hibernation (Beckert et al., 2017) and the truncation of mRNA and deacylated tRNA (Jaishankar and Srivastava, 2017), resulting in smaller, more spherical cells shapes. In *B. subtilis* we detected an enhancement of small (< 2.5 μm) cells at stationary phase (**Figure 1D**), which is further supported by reports of *B. subtilis* producing small (0.7 to 1.15 μm, *n* = 150) round ‘dwarf cells’ that rapidly increase at early stationary phase (Defeu Soufo, 2016). However, in this study, cells 2.5- 5 μm dominated the stationary phase population.

The characteristic rod shape of *B. subtilis* is maintained and elongation occurs *via* the synthesis of peptidoglycan at sites of division and along lateral (non-polar) cell walls (Cabeen and Jacobs-Wagner, 2005). Therefore, cell length was used to determine cell elongation of *B. subtilis* and rod-shaped *L. plantarum.* Elongated chains were observed for *B. subtilis* (**Figure 1D**) and *L. plantarum* (**Figure 2D**) during exponential growth. This exaggerated cellular elongation in *B. subtilis* results from a delay in cell division caused by a metabolic pathway for glucolipid biosynthesis that occurs under high nutrient conditions (Weart et al., 2007; Yao et al., 2012). This type of bacterial growth is typical of inoculation of cells from an overnight culture into fresh media or from cells revived from frozen stock. As demonstrated by *B. subtilis*, elongation during rapid growth allows complete chromosome replication prior to segregation and cell division (Sharpe et al., 1998; Weart et al., 2007). These long chains are therefore a distinct phenotype that can be used to determine rapidly growing cells.

Exponential phase growth of *P. acidilactici* (**Figure 3D**) was characterised by relatively higher proportions of tetrads, compared to pairs of cells and single cells. Although published research on the preferential attachment of cells is scarce, an increased proportion of tetrads presents a distinct phenotype indicative of rapid growth as observed by other Gram-positive bacteria such as the coccoid bacterium *Deinococcus radiodurans* (Slade and Radman, 2011).

A transition to a small cell state during stationary phase is mirrored by *B. subtilis*, *L. plantarum* and *P. acidilactici*, as nutrients become depleted and/or media becomes acidified. The stationary-phase physiology of bacterial cells typically exhibit reduction in the cell wall fluidity and an increase in thickness of the peptidoglycan layer (Jaishankar and Srivastava, 2017). This shift to a small cell size is also demonstrated by other non-spore forming bacteria (van de Guchte et al., 2002), since a small cell has a reduced surface area, compared to elongated cells and is therefore more resilient (Senz et al., 2015). Adaptation to a small cell size has been observed in lactic acid bacteria as a stress response to pH (Narayana et al., 2020) and in glucose depleted media, whereby *L. plantarum* exhibited an increased surface area-to-cell mass ratio, to enable more efficient nutrient uptake (Parlindungan et al., 2018). A small cell state also allows cells to conserve energy (Nystrom, 2004) and is therefore indicative of a survival phenotype.

### The Application of IFC to Distinguish Different Cell Morphologies and Metabolic Activity

In this study, we also show how shifts in cell morphology can be complimented by a variety of fluorescence stains to yield other physiological phenotypic information at cell-level such as cell viability and metabolic activity. We demonstrated the membrane damage and metabolic activity of *L. plantarum* (**Figures 2E, F**) and *P. acidilactici* (**Figures 3E, F**) for individual cells using PI and RSG stains. Minimal cells exhibiting a PI signal reflects the acid tolerant nature of these bacteria, despite the acidification of the growth media (Olajugbagbe et al., 2020). We also classified the morphology of active and inactive cells. In *P. acidilactici*, inactive cells were predominantly, single cells, further supporting the reduction in cell size as an energy saving tactic (**Figures 3G, H**). However, there was no size distinction between metabolically active and inactive cells in *L. plantarum* (**Figures 2G, H**). Interestingly, peaks in mean RSG intensities in stationary phase (as well as exponential phase) may highlight a shift in metabolic activity (**Figures 2B**, **3B**), corresponding to a stress response (Morcrette et al., 2020).

A range of other fluorescence applications can be also performed using IFC, for example monitoring cell morphology and gene expression using fluorescent reporters (Madar et al., 2013). IFC has also been used to quantify the uptake of fluorescently labelled bacteria by host white blood cells (Smirnov et al., 2017) and quantify (fluorescence based) colocalization of pathogens within host cells (Haridas et al., 2017).

Unlike *L. plantarum* and *P. acidilactici*, *B. subtilis* sporulates in response to starvation and increases its cell density, whereby small, resistant, dormant endospores are produced (Pavlendová et al., 2007; Tan and Ramamurthi, 2014), measuring 1.40 ± 0.14 µm in length and 0.55 ± 0.035 µm in diameter, as revealed by scanning transmission electron microscopy (Leuschner and Lillford, 2000; Pavlendová et al., 2007; Tan and Ramamurthi, 2014). Endospores remain dormant until favourable nutrient conditions trigger their germination (Errington and Wu, 2017). In the scope of this study we did not determine whether the observed shift in small cells < 2.5 µm represented spores, however, cells can potentially be discriminated from spores *via* IFC with the application of DNA staining (Karava et al., 2019).

The high-resolution temporal changes in cell phenotypes we show in this study, provides useful insights into how and when cells adapt and respond to external stresses. To the best of our knowledge, this is the first application of IFC to quantify the changing morphology of bacterial cells as they transition from exponential to stationary growth phases. Understanding the morphology of pathogenic bacteria is especially important since the size, shape, aspect ratio and surface properties of pathogens influence their potential for uptake by host cells, determines how the immune system responds to pathogens and, *via* adaptation, allows pathogens to evade detection by immune systems (van Teeseling et al., 2017; Baranov et al., 2021).

### Application of IFC to Determine Cell Concentration

The observed changes of cell shape and size seen in this study and revealed by IFC highlights the acknowledged limitations of standard microbiology techniques for quantifying bacterial cell dynamics. Optical density, for example, is widely used as a rapid tool to measure bacterial growth that is based on the light scattering properties of bacterial populations to determine cell density, from which cell concentrations are inferred. However, the ability of cells to scatter light is dependent on cell shape as well as sample turbidity. Furthermore, OD_600_ measurements are typically used to normalise against other measurements such as fluorescence intensities when investigating florescence reporters to monitor gene expression in cells (Beal et al., 2018). However, the shape and size of bacteria are not uniform, between species and, as shown for each bacterium investigated in this study, cell morphology is a dynamic physiological feature. This ultimately influences inter-species and intra-species OD_600_ measurements.

Single-cell analysis techniques such as conventional flow cytometry offer rapid, high-throughput analysis of individual bacterial cells and provide forward and side scatter information, from which cell size can be inferred. However, some studies report conflicting results from flow cytometry and comparisons with independent imaging of bacterial cells, (Narayana et al., 2020). Cell size can also be rapidly determined using light scattering particle analysers (Üçok and Sert, 2020) or Coulter counters (Senz et al., 2015) however, morphological definition is typically restricted to cell volume. Although microfluidic techniques yield high-resolution imaging of the growth of individual cells and small bacterial populations, (Balaban et al., 2004; Windels et al., 2019) it may not identify rare phenotypes within a wider population.

In contrast, IFC is a high-throughput tool for high-resolution analysis of bacterial phenotypes at cell-level that incorporates cell visualisation, for substantial populations (Madar et al., 2013; Jenner et al., 2016), allowing the characterisation and quantification of both dominating phenotypes and discrete subpopulations such as persister cells.

### Application of IFC to Determine Dormant Cell Types

In this study, we used IFC to identify and track dormant phenotypes of *E. coli*, that had been exposed to ampicillin treatments (**Figures 4**, **6**). Morphological features such as size and side scatter intensity enabled discrimination of lysed cell debris, and PI fluorescence determined dead or damaged cells (**Figure 5**). The resulting persister group, comprised of VBNC and persister cells, displayed a range of cell lengths. However, persister cells, a rare sub-population within the persister group, were determined from the dormant VBNC subpopulation by tracking subtle changes in cell concentration during revival. IFC revealed that persister cells had a discrete size range below 3 µm and detected the growth of these cells over 8 h into longer chains (**Figures 6C–K**). On exposure to ampicillin, *E. coli* has been reported to produce small persister cells (Uzoechi and Abu-Lail, 2020). This small state allows cells to conserve their energy and minimise their surface area, to reduce antibiotic contact (Nystrom, 2004; Uzoechi and Abu-Lail, 2020).

Whilst persister cells have also been shown to exhibit relatively small cell states in wild type *E. coli* (Uzoechi and Abu-Lail, 2020), filamentous persister populations have been detected in ampicillin-tolerant *E. coli*, isolated *via* adaptive laboratory evolution (Sulaiman et al., 2020). In previous studies, the detection and quantification of *E. coli* VBNC and persister cells when cultures were exposed to ampicillin treatments was achieved using traditional flow cytometry (Mohiuddin et al., 2020). A recent study on *Vibrio parahaemolyticus* VBNC cells employed IFC to help distinguish heterogeneous VBNC cell population that were not distinguishable by flow cytometry alone. The use of IFC allowed the authors to first identify sub-populations by imaging and SSC profiles. The authors then combined this with traditional fluorescently activated cell sorting to collect different cell morphological populations and show further differences in proteome profiles (Wagley et al., 2021). This further highlights the importance of flow cytometry data to be visually confirmed. Since IFC allows cells to be visualised and provides detailed morphological analyses, persister cells can be classified with high-resolution and to a hitherto unachievable level of detail that is crucial for understanding antibiotic resistance and relapses in infection (Fisher et al., 2017).

Although multidrug resistant and clinical bacterial strains were not used in this study, our approach to determine cell shape using IFC offers many potential benefits in clinical settings whereby morphology and persister or VBNC behaviour is important. For example, understanding roles of bacterial shape during invasion and colonisation of hosts (Schwartz et al., 2011), morphogenetic factors in pathogenesis (van Teeseling et al., 2017), the potential for using cell shape for targeted drug treatment (van Teeseling et al., 2017), identifying morphological cell changes induced by antibiotic treatments (Cushnie et al., 2016) and understanding the virulence of pathogens with a VBNC state (Li et al., 2014).

In summary, we demonstrate how IFC can be used as a quantitative, high-throughput tool for monitoring cell morphology and identifying discrete, yet diverse bacterial phenotypes within heterogeneous populations. IFC allows the detection of phenotypes that may otherwise not be recognised using traditional techniques such as optical density, CFU counts, microfluidics and conventional flow cytometry. We demonstrate a template for determining cell morphology that could be widely applied to a range of bacteria, including pathogenic species, to further understand morphological traits and the implications of cell shape in a range of microbiological applications. Furthermore, IFC can be used to deepen our understanding cell phenotypes during cell cycles, identifying dormant (VBNC) cells in response to environmental conditions, or assessing the performance (product formation) of bacteria used in biotechnological applications.

## Data Availability Statement

The original contributions presented in the study are included in the article/**Supplementary Material**. Further inquiries can be directed to the corresponding authors.

## Author Contributions

AP, SW, DB, SG, and JL contributed to the conception and design of the study. AP and DB acquired data. AP analysed and interpreted data. AP performed statistical analysis, constructed figures and wrote the first draft of the manuscript. AP and SW wrote sections of the manuscript. All authors contributed to the article and approved the submitted version.

## Funding

This study received funding from Shell International Exploration and Production Incorporated. The funder was not involved in the study design, collection, analysis, interpretation of data, the writing of this article or the decision to submit it for publication. All authors declare no other competing interests.

## Conflict of Interest

Authors PL and DP are employed by Shell International Exploration & Production 578 Incorporated.

The remaining authors declare that the research was conducted in the absence of any commercial or financial relationships that could be construed as a potential conflict of interest.
