# Supplementary material for: The Application of Imaging Flow Cytometry for Characterisation and Quantification of Bacterial Phenotypes
Source: Front Cell Infect Microbiol. 2021 Jul 21;11:716592. doi: 10.3389/fcimb.2021.716592 (PMC8335544; doi:10.3389/fcimb.2021.716592)
Supplement: Supplementary file 1 [file DataSheet_1_v1.pdf]

*Supplementary Material*

# The application of Imaging Flow Cytometry for characterisation and quantification of bacterial phenotypes

## 1 Supplementary Figures

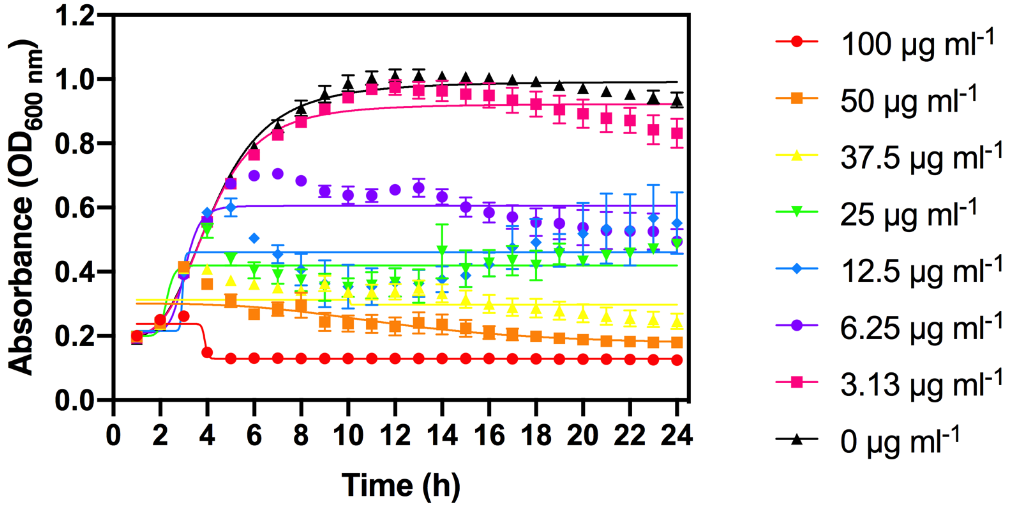


**Supplementary Figure 1: Determining minimum inhibitory concentrations of ampicillin in *E. coli DH5α*.** Dose response curves of varying ampicillin concentrations are presented. We determined that 6.25 µg ml $^{-1}$ was the minimum inhibitory concentration. $n$ = 3, error bars represent standard error of the mean.

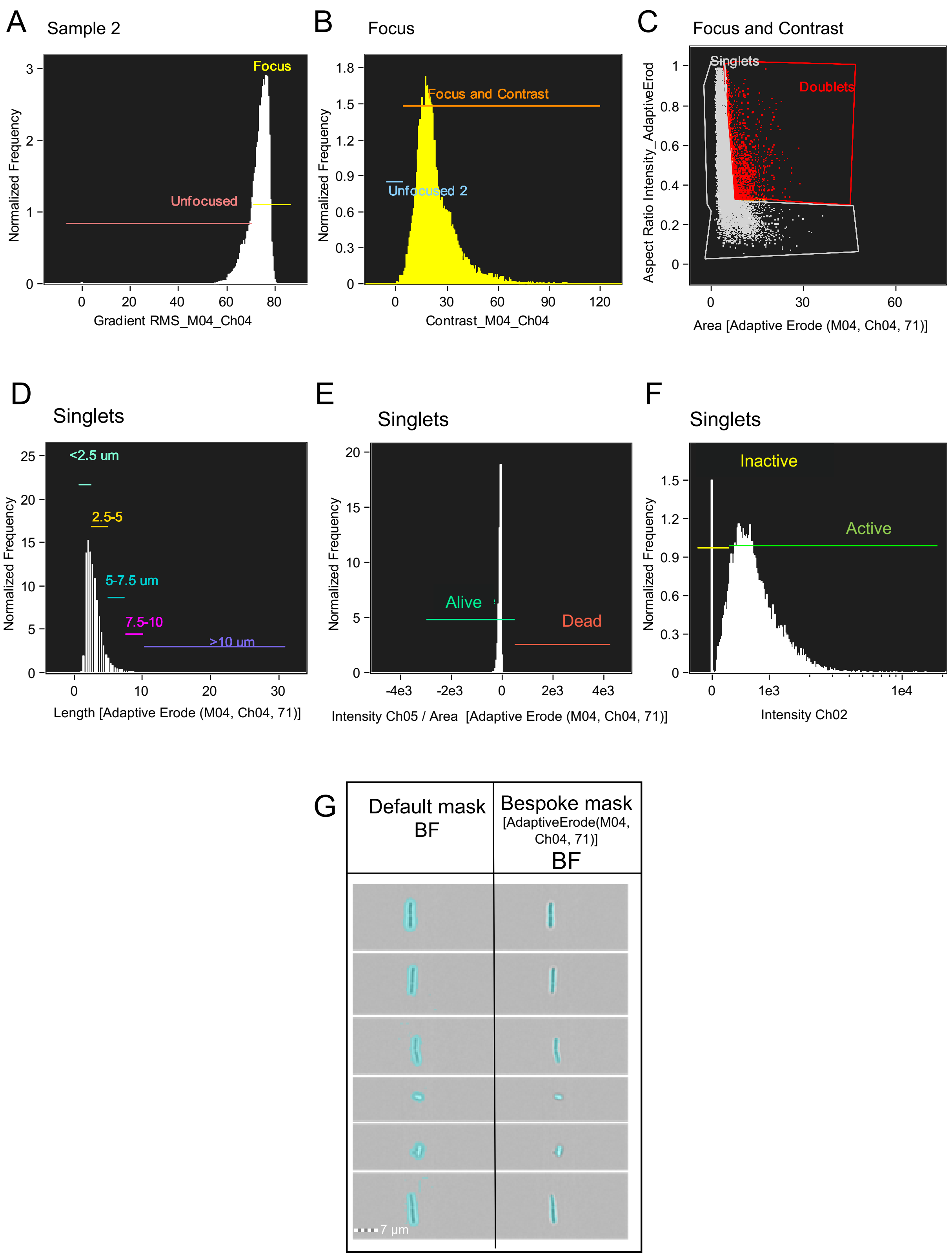


**Supplementary Figure 2: Imaging flow cytometry gating strategy used to concentrate single, focused cells, and mask creation to ensure accurate morphological data: An example from *L. plantarum* WCFS1 experiment.** Gradient RMS of Brightfield (BF) default mask (M04, Ch04) was

used to initially discern between focused ‘Focus’ and ‘Unfocused’ cells (A). Contrast (M04, Ch04) was used to further exclude unfocused cells ‘Unfocused 2’ (B). Area and aspect ratio features were applied to identify ‘Doublets’ (multiple object image captures) from ‘Singlets’ (individual cell image captures) (C). The ‘Singlets’ population was then classified based on BF cell length at 2.5 µm intervals (D). ‘Propidium Iodide signals, indicating cell membrane damage, were detected via red fluorescence (Ch05: 702/85 nm) intensity, normalised for cell area (Supplementary Figure 3). Metabolic activity, as indicated by Redox Sensor Green was detected via green fluorescence (Ch02: 533/55 nm) intensity (Supplementary Figure 4).

*L. plantarum*

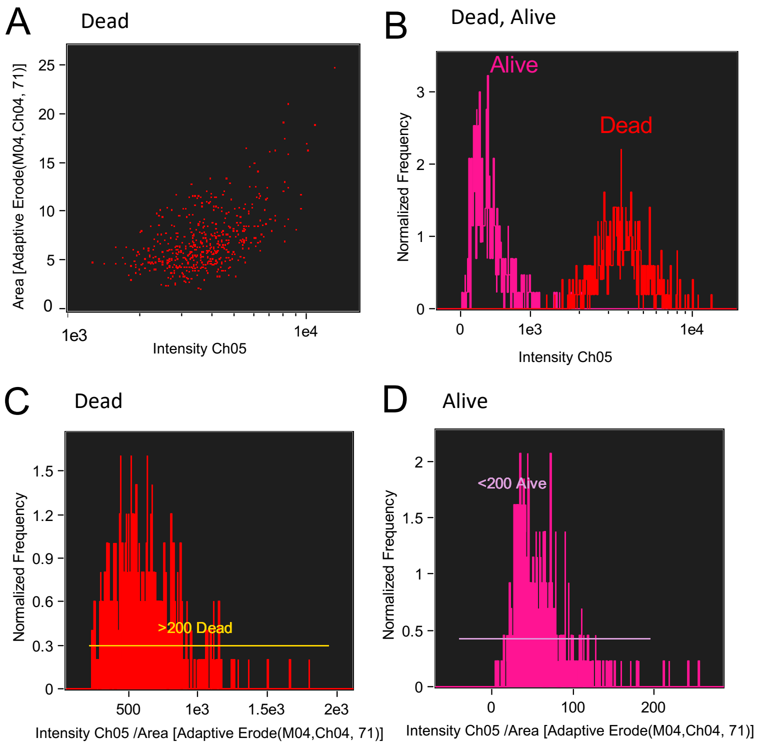


*P. acidilactici*

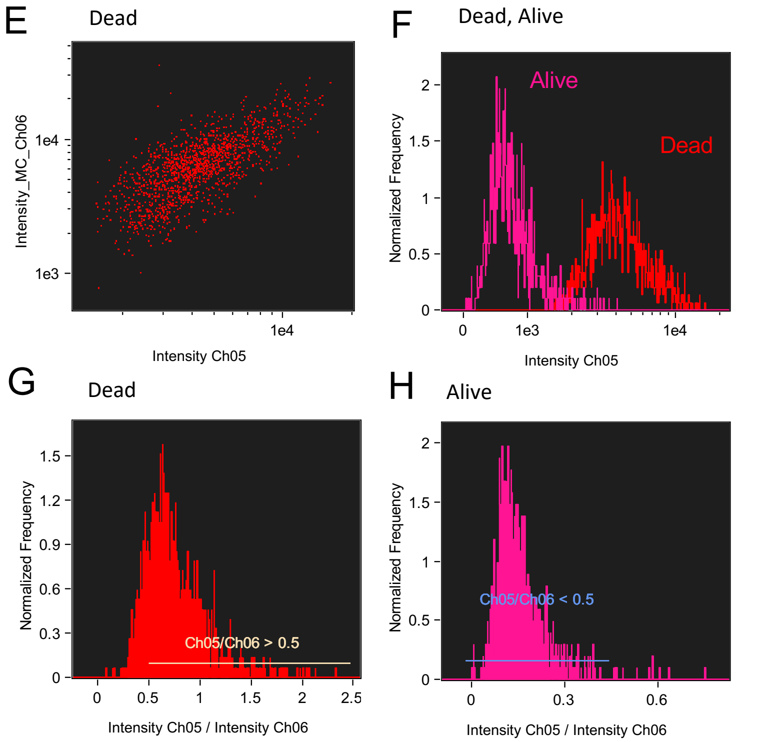

**Supplementary Figure 3: Determination of PI signal, normalised for cell size: examples from *L. plantarum* WCFS1 and *P. acidilactici*.** Propidium iodide signals indicate a compromised cell wall. *L. plantarum* WCFS1 and *P. acidilactici* cultures were grown to mid-log (negative control: 'alive') and a sample of these mid-log cells were exposed to a lethal temperature (90 °C) for 30 minutes and represent 'dead' cells (positive control). Samples were stained with Propidium iodide. A mixed population of alive and dead cells (50:50 % by volume) was then analysed. Out of focus cells were excluded since unfocused cells can exhibit lower fluorescence signal strength. An increase in PI intensity was observed with side scatter intensity (A and E), therefore, to accurately determine cells exhibiting a true PI signal, Ch05 (702/85 nm) intensity (B and F) was normalised for cell size. For *L. plantarum* WCFS, Ch05 intensity was normalised for cell area (brightfield) and a Ch05/area ratio of BF >200 was adopted to identify cells with a positive PI signal (C and D). For, *P. acidilactici* Ch05 intensity was normalised by SSC intensity, due to the coccoid nature of cells and difficulty distinguishing cell size based solely on brightfield images. Ratios > 0.5 indicative of cells with a positive PI signal (G and H).

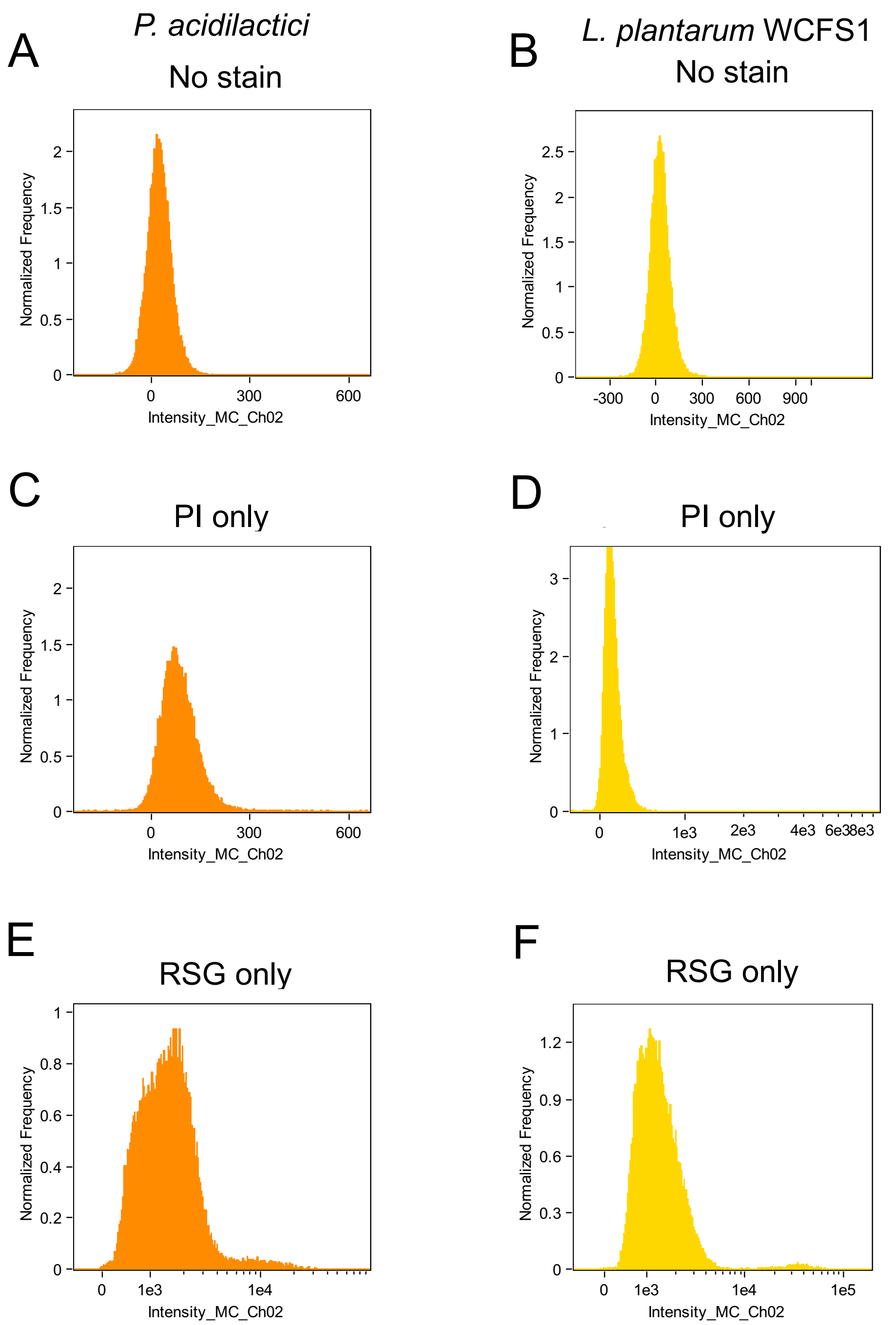


**Supplementary Figure 4: Determination of Redox Sensor Green (RSG) signals at cell-level in *P. acidilactici* and *L. plantarum* WCFS1 via identification of background fluorescence.** *L. plantarum WCFS1* exhibits Ch02 (533/55 nm) intensity values < 300 when no stains are applied (A) and when only propidium iodide (PI) is applied (C). *P. acidilactici* has a slightly lower background Ch02 intensity values of <200 when no stain is applied (B) and when PI is applied (D). Therefore, thresholds of >200 and >300 for Ch02 intensity were selected to identify cells exhibiting a positive RSG signal for *P. acidilactici* (E) and *L. plantarum* WCFS1 (F), respectively.

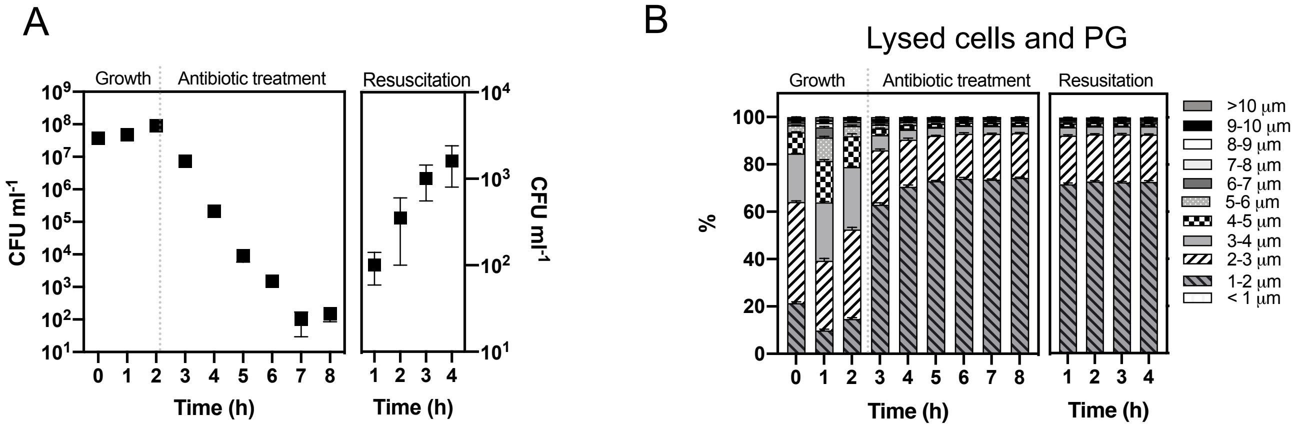


**Supplementary Figure 5: Tracking cell phenotypes in *E. coli* during initial 2 h growth, 6 h (short-term) antibiotic treatment and 4 h resuscitation period.** Colony forming Unit (CFU) counts were used to monitor bacterial growth (A). Imaging flow cytometry was used to track proportion of cell sizes (B). Low CFU counts and minimal changes in the size dynamics of *E. coli* during 4 h resuscitation period reflects minimal detectable growth. n = 4, error bars represent standard error of the mean.

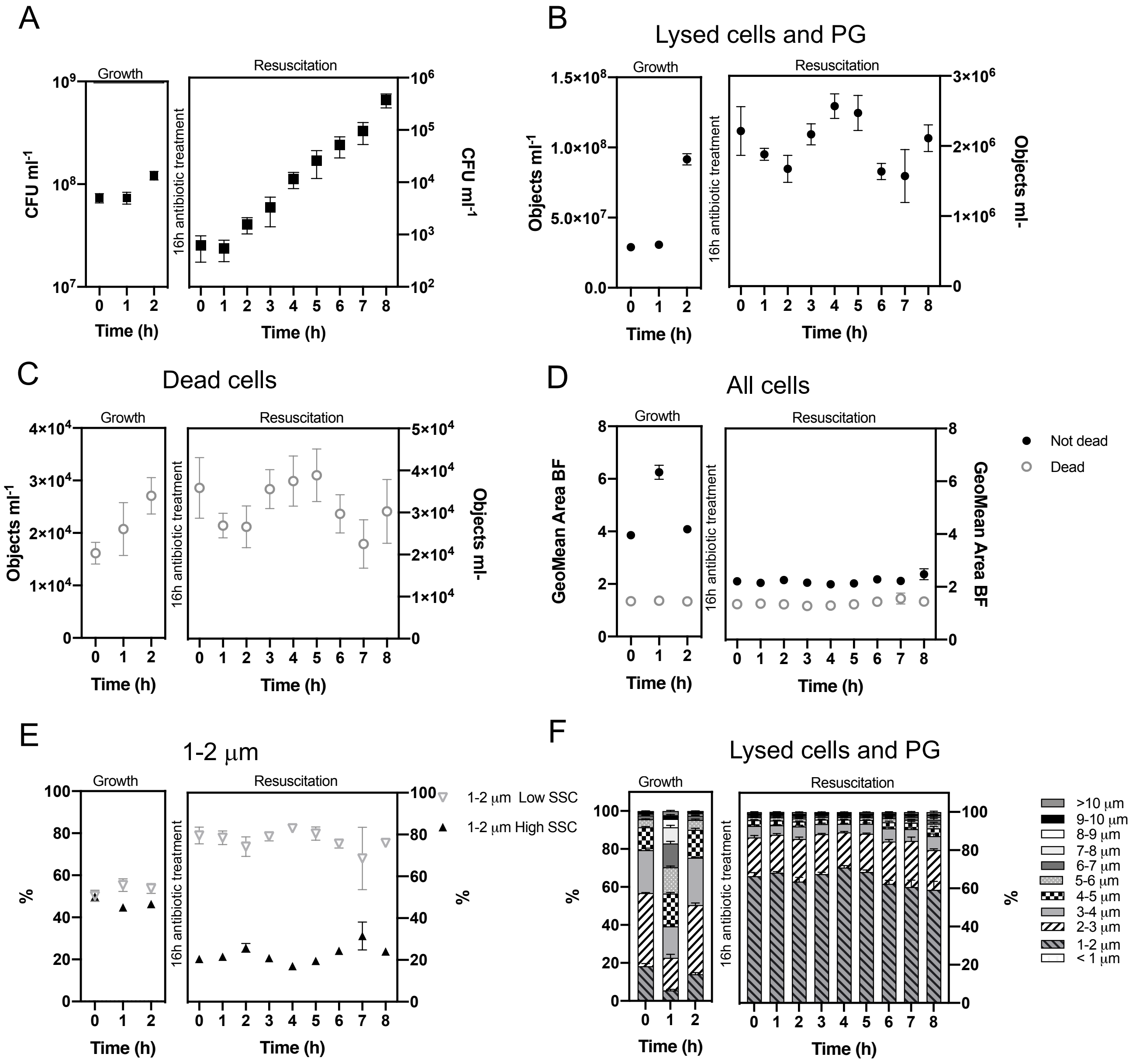


**Supplementary Figure 6: Tracking cell phenotypes in *E. coli* during initial 2 h growth, and 8 h resuscitation period following long-term (16 h) antibiotic treatment.** Colony forming unit (CFU) counts were used to monitor bacterial growth (A). Imaging flow cytometry was used to track cell concentrations (objects $ml^{-1}$) of Total cells (B) and Dead cells (C). A reduction in cell size occurs with exposure to antibiotic and is sustained throughout the resuscitation phase (D). The concentration of cells < 2 um with low side scatter intensities (E) and the proportion of cell sizes (F) were monitored throughout growth and resuscitation. n = 5, error bars represent standard error of the mean.

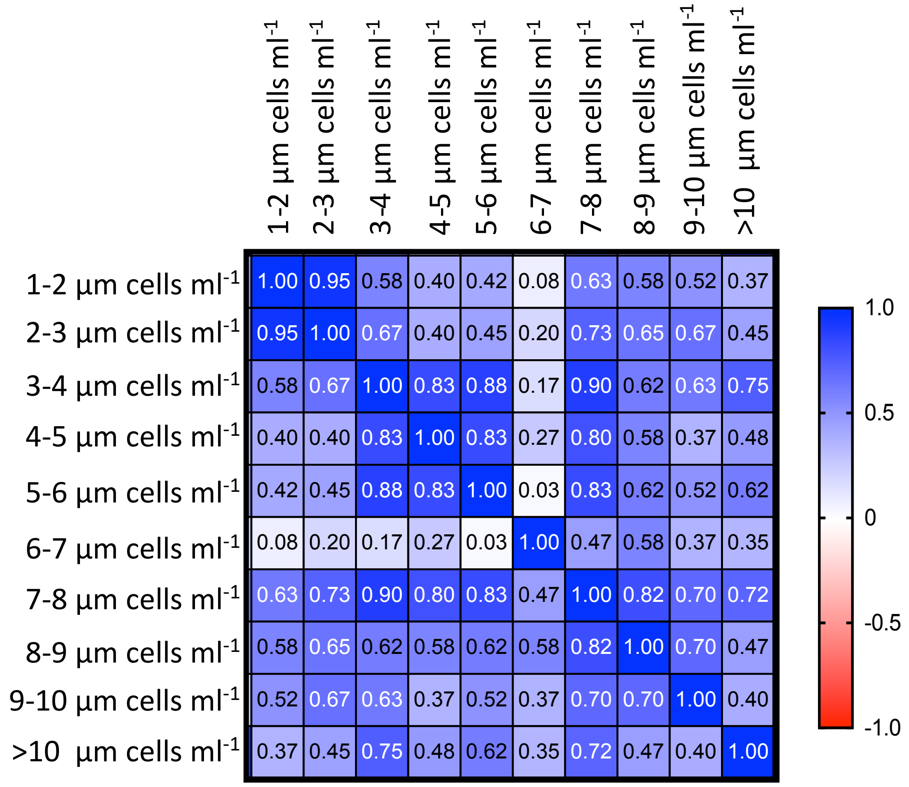


| | 1-2 µm cells ml$^{-1}$ | 2-3 µm cells ml$^{-1}$ | 3-4 µm cells ml$^{-1}$ | 4-5 µm cells ml$^{-1}$ | 5-6 µm cells ml$^{-1}$ | 6-7 µm cells ml$^{-1}$ | 7-8 µm cells ml$^{-1}$ | 8-9 µm cells ml$^{-1}$ | 9-10 µm cells ml$^{-1}$ | >10 µm cells ml$^{-1}$ |
|---|---|---|---|---|---|---|---|---|---|---|
| 1-2 µm cells ml$^{-1}$ | 1.00 | 0.95 | 0.58 | 0.40 | 0.42 | 0.08 | 0.63 | 0.58 | 0.52 | 0.37 |
| 2-3 µm cells ml$^{-1}$ | 0.95 | 1.00 | 0.67 | 0.40 | 0.45 | 0.20 | 0.73 | 0.65 | 0.67 | 0.45 |
| 3-4 µm cells ml$^{-1}$ | 0.58 | 0.67 | 1.00 | 0.83 | 0.88 | 0.17 | 0.90 | 0.62 | 0.63 | 0.75 |
| 4-5 µm cells ml$^{-1}$ | 0.40 | 0.40 | 0.83 | 1.00 | 0.83 | 0.27 | 0.80 | 0.58 | 0.37 | 0.48 |
| 5-6 µm cells ml$^{-1}$ | 0.42 | 0.45 | 0.88 | 0.83 | 1.00 | 0.03 | 0.83 | 0.62 | 0.52 | 0.62 |
| 6-7 µm cells ml$^{-1}$ | 0.08 | 0.20 | 0.17 | 0.27 | 0.03 | 1.00 | 0.47 | 0.58 | 0.37 | 0.35 |
| 7-8 µm cells ml$^{-1}$ | 0.63 | 0.73 | 0.90 | 0.80 | 0.83 | 0.47 | 1.00 | 0.82 | 0.70 | 0.72 |
| 8-9 µm cells ml$^{-1}$ | 0.58 | 0.65 | 0.62 | 0.58 | 0.62 | 0.58 | 0.82 | 1.00 | 0.70 | 0.47 |
| 9-10 µm cells ml$^{-1}$ | 0.52 | 0.67 | 0.63 | 0.37 | 0.52 | 0.37 | 0.70 | 0.70 | 1.00 | 0.40 |
| >10 µm cells ml$^{-1}$ | 0.37 | 0.45 | 0.75 | 0.48 | 0.62 | 0.35 | 0.72 | 0.47 | 0.40 | 1.00 |

**Supplementary Figure 7: Spearman's Rank correlation coefficient matrix used to deterine statistically significant relationships between cell size classifications of *E. coli* during 8 h resuscitation period, following long-term (16 h) ampicilin treatment.** R values > 0.8 sigifiying a p-value < 0.01 were identified as significant.
